# Long term influence of groundwater preservation policy on stubble burning and air pollution over North-West India

**DOI:** 10.1038/s41598-022-06043-8

**Published:** 2022-02-08

**Authors:** Yogesh Kant, Prakash Chauhan, Aryan Natwariya, Suresh Kannaujiya, Debashis Mitra

**Affiliations:** Indian Institute of Remote Sensing (IIRS), ISRO, Department of Space, Govt. of India, 4-Kalidas Road, Dehradun, India

**Keywords:** Sustainability, Environmental impact

## Abstract

Stubble burning (SB) has been a major source of seasonal aerosol loading and pollution over northern India. The aftereffects of groundwater preservation act i.e., post 2010 era (2011–2020) has seen delay in crop harvesting thereby shifting the peak SB to May (Wheat SB) and to November (Paddy SB) by 8–10 and 10–12 days compared to pre-2010. Groundwater storage depletion rate of 29.2 mm yr^−1^ was observed over the region. Post 2010 era shows an increase of 1.4% in wheat SB and 21% in Paddy SB fires over Punjab and Haryana with 70% of PM_2.5_ air mass clusters (high probability > 0.8) advecting to the downwind regions leading to 23–26% increase in PM_2.5_ and 4–6% in aerosol loading over National Capital Region (NCR). Although the objective of water conservation policy was supposed to preserve the groundwater by delaying the paddy transplantation and sowing, on the contrary the implementation of this policy has seen groundwater storage after 2013 depleting at a rate of 29.2 mmyr^−1^ over these regions. Post policy implementation has led to shift and shrinking of harvest window with increased occurrences in SB fires which also increase associated particulate matter pollution over North India.

## Introduction

About 80% of the total global Biomass Burning (BB) is contributed by tropical regions, mainly Southeast Asia, Southern Africa, Australia and South America^1^. About one fourth of worldwide BB events are due to Stubble Burning (SB), whereas SB over Asia is 34% (forest fires 45% and grassland fires 20%)^2,3^. About 44% of SB is contributed by China and 33% is contribution from Indian farms^2^. It is estimated that a total of 620 million tons of crop residue is generated annually in India, of which 16% is burnt in field and major contribution in SB is paddy straw (43%), wheat straw (21%) followed by sugarcane (19%)^3^. Punjab accounts for 21.32 million tons (mt) stubble burnt every year (out of 51 million ton of crop residue) while Haryana 9.18 mt (out of 28 mt crop residue)^3^. Over the last 15 years, the total production of wheat & paddy over Punjab and Haryana has increased by 6.96% while the rate of surplus crop residue left over in the field (after utilization for cattle fodder, storage & other uses)^4^ is 1.2 mt yr^−1^. Surplus rate of crop residue will continue to increase if substantive mechanism for disposal of crop stubble or other alternatives are not provided to farmers (85–90% of this paddy straw is burnt in the field^5^). Satellite data shows that the stubble burning in increasing in recent years^6^. It is estimated that SB fires have increased at a rate of 250 per year over Punjab and Haryana during 2003–2017^6^. Despite the government policies prohibiting ways to tackle stubble burning and groundwater preservation (Prevention and Control of Pollution Act 1981; The Punjab Preservation of Subsoil Act 2009; The Haryana Preservation of Subsoil Act 2009 (hereafter GW acts); National Policy for Management of Crop Residue 2014; National Green Tribunal 2015), the practice is still continuing. Various studies show that the groundwater over Punjab and Haryana has depleted even after the implementation of 2009 water preservation act at an alarming rate^7,8^. A net loss of around 109 km^3^ during 2002–2008 using NASA’s GRACE^9^ which is as a result of changing cropping pattern and usage of ground water for irrigation. In Punjab, the number of tube wells have increased to 1.47 million in 2019 (compared to 0.19 million in 1970) while observed groundwater level reduced by 8.91 m averaged for Punjab state from 2000–2019^8^. Various studies have proven that SB adversely effects the air quality and thereby human health^10,11^. The economic and health costs of air pollution caused due to stubble burning in North India is around $30 billion per year^12^. The impact of pollution (particularly PM_2.5_ levels) has increased over North India in the recent times^13^ (till 2015) and in post-monsoon in Delhi due to the effect of SB^14^. On an average ~ 20% of total PM_2.5_ concentration observed in Delhi during October and November (post-monsoon) is contributed by external advection and about 50–75% PM_2.5_ concentration is contributed during the SB period from the upwind region^15^. It is estimated that 66,200 PM_2.5_ attributable deaths (6.1%) are caused by stubble burning in India in 2015^16^. Introduction of post 2009 GW act, has observed a 10-day shift in timing of post-monsoon SB over NW India^17^ and its influence on surface air pollution (PM_2.5_) over Delhi is estimated ranging between 7–78% of the maximum observed PM_2.5_ enhancements in Delhi to fires during post-monsoon season^18^. We investigated the impact of GW act on the changes & pattern in stubble burning, its impact on air pollution in the region and status of groundwater storage thereafter.

## Results

### Long term variation in stubble fires

**Figure 1:**
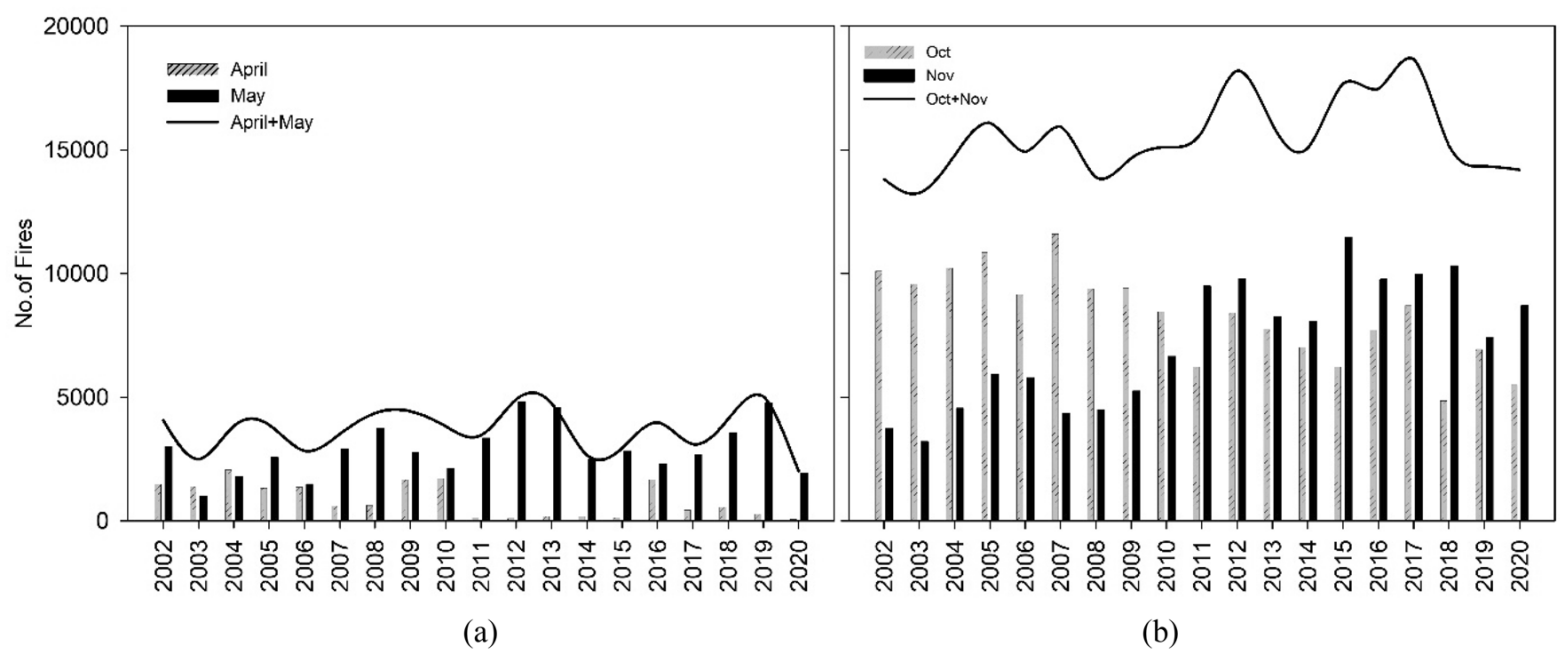
Time series of monthly fire counts for (**a**) PrM_SB_ and PoM_SB_ (**b**) during 2002–2020.

Punjab and Haryana which accounts to 2.87% of India’s landmass is situated in northwestern part of Indo-Gangetic Plain (IGP) (Supplementary Fig. 1a). Over Punjab and Haryana Wheat stubble is burned in April & May pre-monsoon season (PrM_SB_) and Paddy stubble is burned in October & November post-monsoon season (PoM_SB_). During 2002–2020, the average number of SB observed over Punjab and Haryana during PrM_SB_ and PoM_SB_ are 3585 and 15,972 per yr^−1^ respectively (Fig. 1a,b). From satellite based daily fire counts, it is observed that average PoM_SB_ (~ 16,000 fires yr^−1^) are 4–5 times higher than the PrM_SB_ (~ 3600 fires yr^−1^). The non-parametric Mann–Kendall test shows a significant increasing trend with a rate of 0.21% yr^−1^ and 1.51% yr^−1^ (P < 0.05) for PrM_SB_ and PoM_SB_ respectively during 2002–2020. Over the past 19 years, paddy stubble fires have increased by 36% (245 fires yr^−1^) as compared to wheat stubble fires of 4% (15 fires yr^−1^) (Fig. 1a,b). Post-2010 era, witnessed PoM_SB_ fires increased by 21% (compared to pre-2010 era) while PrM_SB_ fires increased by 1.4% (compared to pre-2010 era). Monthly fire counts of post-2010 era, reveal that the April SB fires have decreased (0.4% yr^−1^) and fires in May have increased (0.65% yr^−1^) while decrease of stubble fires in October (2.1% yr^−1^) and a significant jump in November (3.2% yr^−1^) (Fig. 1a,b). This clearly establishes the fact that the distribution of majority stubble fires after year 2010 has shifted from April to May and substantial shift from October to November.

**Figure 2:**
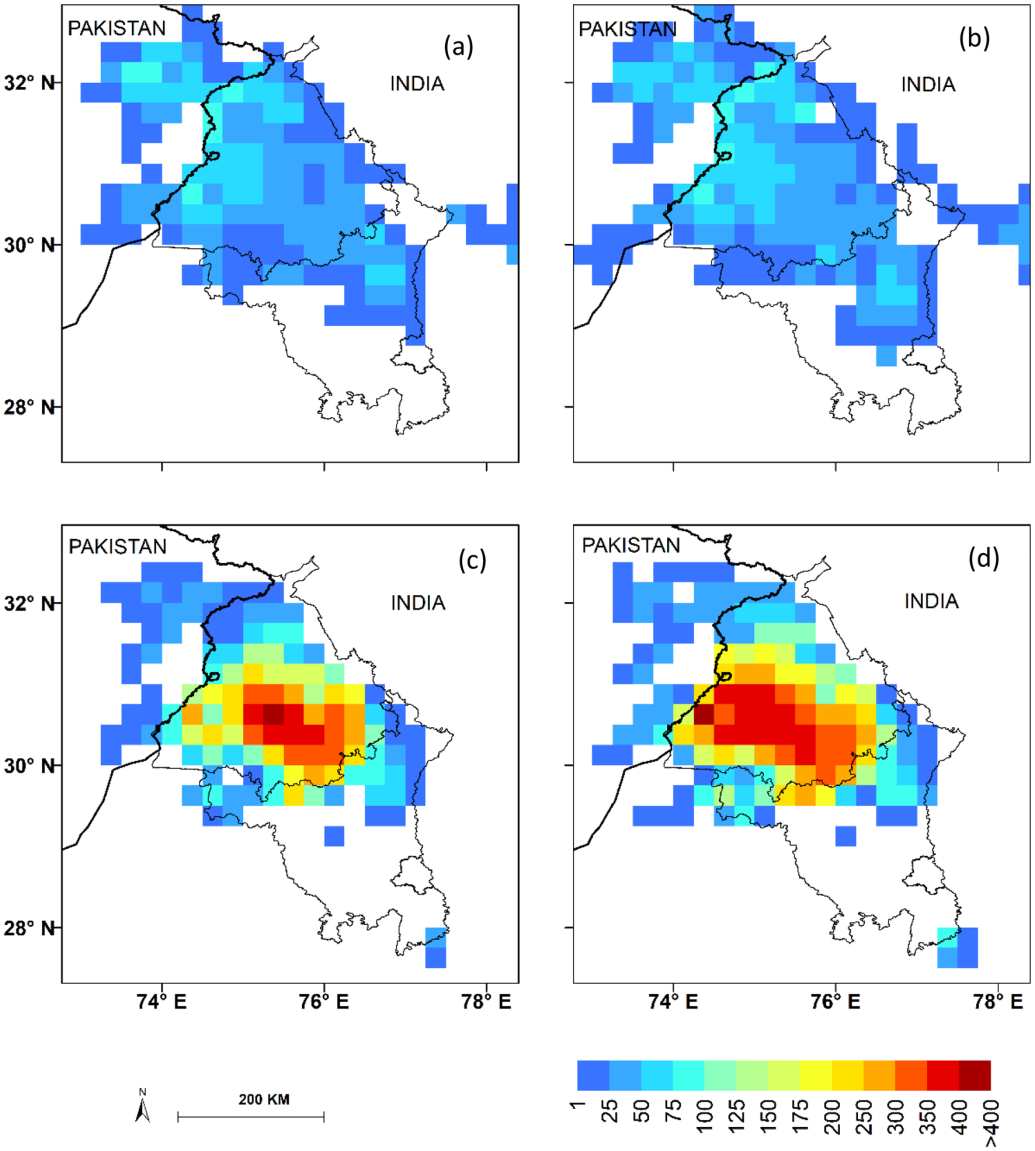
Fire density (no. of fires per day at 0.25 × 0.25° grid) for pre-2010 era (left panel) & post-2010 era (right panel); (**a**,**b**) PrM_SB_ and (**c**,**d**) PoM_SB_, respectively. The maps are generated using licensed software ArcGIS version 10.6.

Fire density helps in understanding the spatial distribution, variation and intensity of SB fires (Fig. 2). The fire density has been classified as High Fire Prone region (HFP) (fire density > 300), Moderate Fire Prone region (MFP) (150–300 fire density) while Low Fire Prone region (LFP) (50–150 fire density). It is observed that during PrM_SB_, maximum fire density is in LFP zone which has increased by 21% while during PoM_SB_ the hotspot region under HFP has increased by ~ 51% while MFP region by 32% from pre-2010 era (Fig. 2a–d). This is clear indication that in-spite of the enforcement of the GW act (post-2010 era) there is relatively marginal increase in fire occurrences in PrM_SB_ while substantial jump in PoM_SB_ burning, implying that the stubble burning practice is continuing. This increase and peak shift of burning pattern (1st week of November) in PoM_SB_ season coincide with the festive season in India with high anthropogenic carbonaceous dust, smoke and aerosol particulate matter (PM_2.5_, PM_10_) adversely affecting air quality and are advecting to regions of National Capital Region (NCR) and parts of Indo-Gangetic Plain.

### Shift in pattern of stubble fires

**Figure 3:**
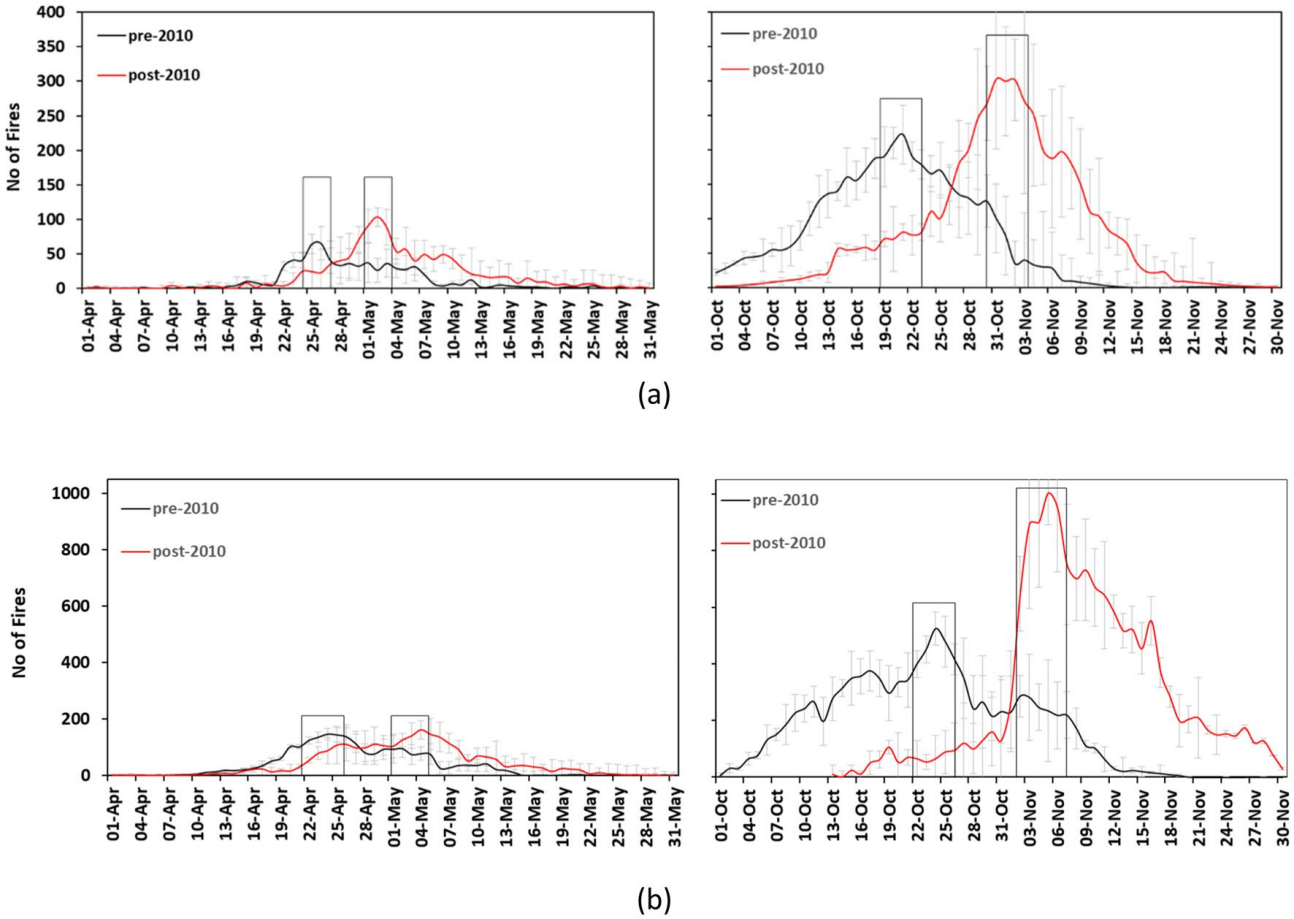
Daily average fire counts for PrM_SB_ (left) and PoM_SB_ (right) along with peak fire shift (in days) over Haryana (**a**) and Punjab (**b**) hotspot region during pre- and post-2010 era.

During pre-2010 era, PrM_SB_ practice over Punjab and Haryana started from 2nd week of April–2nd week of May (~ 31 days) (Fig. 3a,b-left) (with peak time during 24–27 April in Haryana and 23–27 April in Punjab) while PoM_SB_ from 1^st^ week of October—2nd week of November (~ 37 days) (Fig. 3a,b-right) (with peak time during 19–24 October in Haryana, and 22–27 October in Punjab). After the reinforcement of GW act *i.e.,* post-2010 era, the peak of PrM_SB_ shifted from April to May and for PoM_SB_ shifted from October to November (Fig. 3a,b). Now, the length of PrM_SB_ episode is from the 2nd week of April and lasts till 3rd week of May (~ 39 days) and PoM_SB_ window is from 2nd week of October to about 4th week of November (~ 48 days). Peak stubble burning during the post-2010 era was recorded during 1–4 May & 2–6 May and 31st October–4th November & 2–8 November in Haryana and Punjab respectively. Similar PoM_SB_ burning peak during post 2010 era was also observed elsewhere^19^. This clearly establishes that stubble burning events have been shifted by 8–10 days during pre-monsoon and 10–12 days during post-monsoon season in both Punjab and Haryana.

### Status of groundwater storage

**Figure 4:**
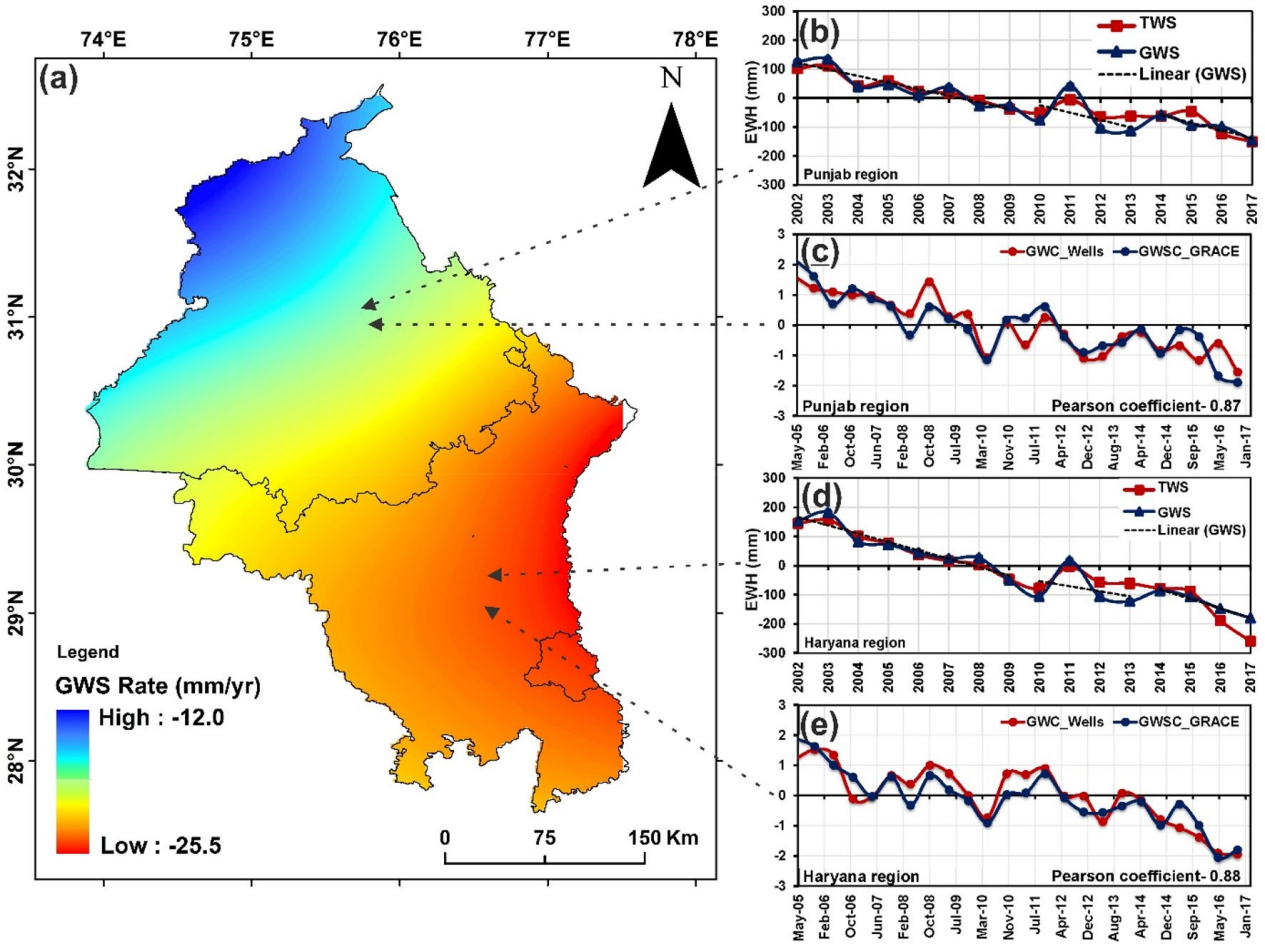
(**a**) The Groundwater storage rate variation in regions of Punjab and Haryana, exhibiting a high rate of depletion in Haryana regions (map is generated using licensed software ArcGIS version 10.6) (**b**,**d**) Time series plotted on TWS and GWS variation in Punjab and Haryana regions, respectively. (**c**,**e**) Validating the estimated GWS calculated from the GRACE dataset with the GWC of the well dataset in Punjab and Haryana region, respectively, gving a Pearson coefficient of 0.87 and 0.88.

The groundwater storage (GWS) depletion rate observed in Punjab is 16.02 $\pm$ 2.1 mmyr^−1^, whereas in Haryana, is  21.5 $\pm$ 2.8 mmyr^−1^ (Fig. 4a,b,d). After the enforcement of the GW act in 2009, we noticed a temporary phase of improvement in groundwater storage change rate during 2010–2013 period (compared to 2002–2009) is + 0.18 and + 8.16 mm yr^−1^. However, after 2014, there is a drastic deterioration in depletion of the GWS change rate at  3.57 and  4.45 mm yr^−1^ over Punjab and Haryana, respectively (compared to 2010–2013 period) (Fig. 4b,d; supplementary table 1). The calculation of groundwater mass reveals that groundwater loss is 1.28 km^3^ yr^−1^ and 0.79 km^3^ yr^−1^ during 2010–2013 (compared to 1.29 km^3^ yr^−1^ and 1.46 km^3^ yr^−1^ during 2002–2009) over Punjab & Haryana. During 2014–2017 period, groundwater loss estimated has increased to 1.32 km^3^ yr^−1^ and 1.46 km^3^ yr^−1^, respectively. The groundwater level rate (m yr^−1^) using the Central Ground Water Board (CGWB) in-situ data also indicate improvement in the groundwater table during 2010–2013 while drastic deterioration (131% & 255% enhancement in depletion rate over Punjab and Haryana) after 2013 (supplementary table 2). These results are further validated with ground well data (135 wells for Punjab and 256 wells for Haryana). A good correlation of 0.88 & 0.87 Pearson coefficient are estimated for Punjab & Haryana regions respectively (Fig. 4c,e). It clearly indicates that both Haryana and Punjab region is facing a groundwater crisis, but groundwater in the Haryana is comparatively in a more water-stressed situation. The average SPI values were calculated using CHIRPS data for Punjab and Haryana region during 2002–2017 period (Supplementary Fig. 2a,b). Based on SPI-6, the historical drought condition for the period 2002–2009 is reflected by SPI values that lie between ~ 0 to − 1.5 which according to Tom Mckee classification falls under moderate drought category over Punjab and Haryana region. While the drought condition eased from 2010–2017 (witnessing promising rainfall compared to pre-2010 era) with SPI values between 1.5 to − 1 indicating moderate wet condition (Supplementary table 3). During 2010–2017, Punjab and Haryana experienced good annual precipitation (50–75 mm) & (50–80 mm) than during 2002–2009 period (30–40 mm) & (30–50 mm) respectively (Supplementary Fig. 2)**.** Inspite of good annual rainfall, the annual GWS rate deteriorated drastically during 2010–2017 indicating excessive usage of groundwater for irrigation.

### Variation of aerosol particulate matter

The climatological average (2002–2020) AOD over NCR during PrM_SB_ and PoM_SB_ is observed as 0.51 ± 0.04 (PrSB_20_ = 0.54 ± 0.05) and 0.62 ± 0.07 (PoSB_50_ = 0.71 ± 0.08) (Supplementary Fig. 3a,b). AOD in post-2010 era witnessed an increase of 9.6% (5.4% in PrM_SB_ & 12.2% in PoM_SB_) (compared to pre-2010 era) while in last 5 years, AOD decreased by 3.1% (0.61% yr^−1^ during 2016–20 compared to 2011–2015 period) (Supplementary Fig. 3). The climatological PM_2.5_ average concentration over NCR during 2011–2020 is 104 ± 17μgm^−3^ during PrM_SB_ (PrSB_20_ = 131 ± 12 μgm^−3^) and 169 ± 26 μgm^−3^ in PoM_SB_ (PoSB_50_= 220 ± 31 μgm^−3^) over NCR has decreased at the rate of 0.27% yr^−1^ and 0.1% yr^−1^ (P < 0.05), respectively (Supplementary Fig. 4a,b). As per data available from CPCB, (supplementary table 4), the suitable data points from 2011, were taken for analysis, as data for pre 2011 period is not continuously available. Over Delhi PM_2.5_ increased by 17.5% (0.92% yr^−1^ in PrM_SB_ & 2.6% yr^−1^ in PoM_SB_) compared to pre-2010 era while last 5 years witnessed decrease of 8.5% (1.67% yr^−1^ during 2016–2020 compared to 2011–2015 period).

**Figure 5:**
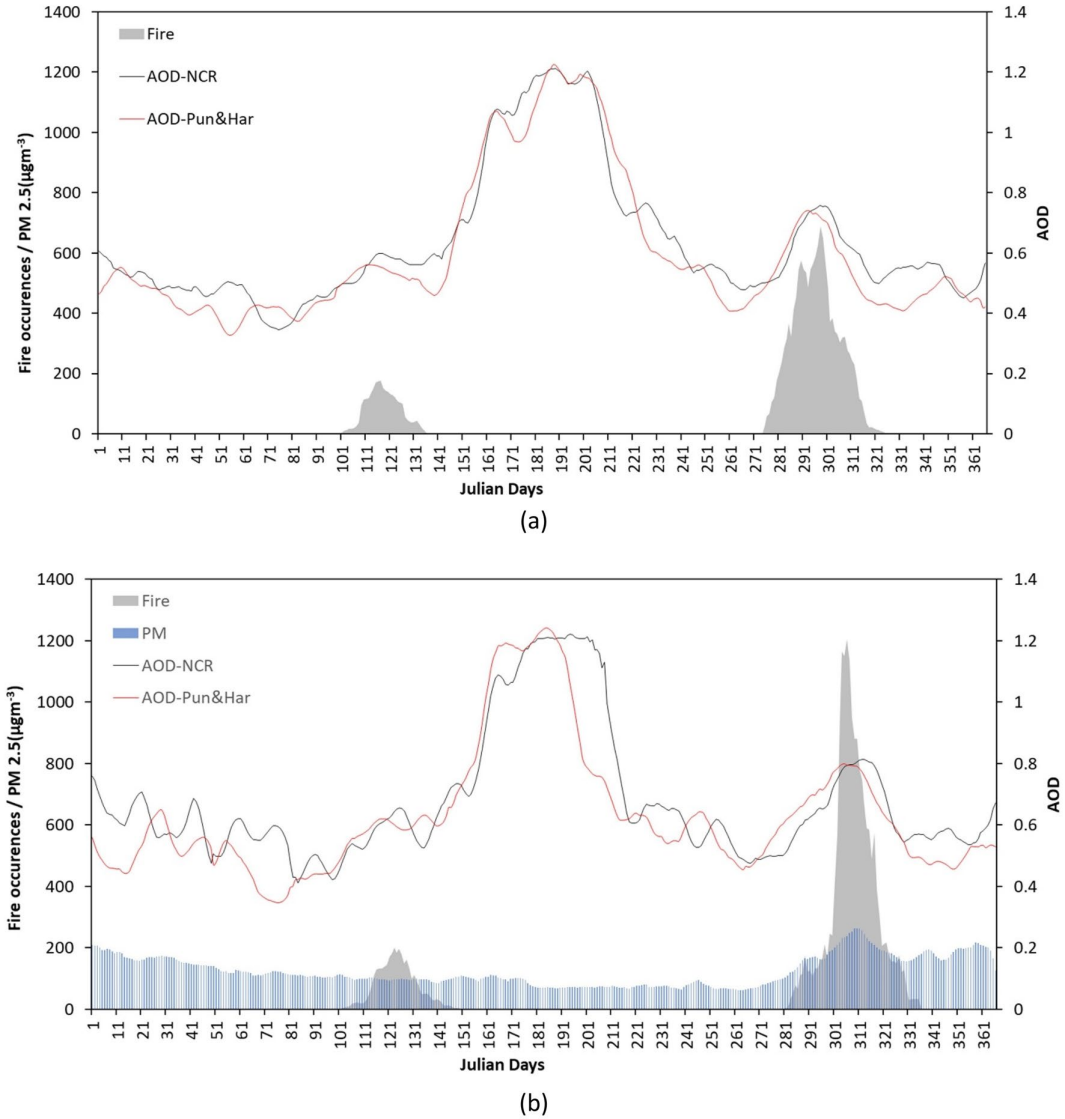
Daily average AOD and PM_2.5_ over NCR and Fire counts over Punjab and Haryana during 2002–2010 (**a**) and 2011–2020 (**b**) (PM_2.5_ data is from 2010–2020) (High AOD over NCR during summer monsoon is attributed to thin and moving clouds prevailingduring the period).

In order to understand the relationship of the SB fires to AOD and PM_2.5_, its daily average variation during 2010–2020 over NCR region was analyzed (Fig. 5). The background emissions particularly PM_2.5_ in last six years have reduced due to implementation of several policies like Graded Response Action Plan (GRAP), Comprehensive Action Plan (CAP), and National Clean Air Programme (NCAP) for prevention, control and mitigation of air pollution in Delhi and NCR^20^. This also resulted in a decrease in the number of days that exceeds the Indian National Assessment Quality Standards in and around Delhi and its nearby cities^21^. Further, 1457 out of 1542 coal and oil-driven industries have shifted to greener fuel, compressed natural gas (CNG) in New Delhi^22^. The annual growth of vehicles in Delhi decreased from 8.13% in 2005–2006 to 3.69% 2018–2019^23^ and registration of commercial CNG vehicles increased by 54% during 2011 to 2015^24^. As such there is negligible increment in rate of background emissions in NCR during the SB period and as biomass burning is prohibited in Delhi, the only external factor influencing the NCR environment is dust/smoke originating from hotspot region (Punjab, Haryana) during PrM_SB_ (110–159 Julian day) and PoM_SB_ (280–330 Julian day) including mineral dust storm from far west (high AOD but low Black Carbon) during (− 160–185 Julian day). It is evident that during pre-2010 era (Fig. 5a), AOD during PrM_SB_ period over Punjab & Haryana peaks during 111 JD which takes to peak over NCR region around 116 JD while in post-2010 era (Fig. 5b) AOD over Punjab & Haryana peaks around 118 JD which takes to peaks over NCR around 124 JD. Similarly, during PoM_SB_ pre-2010 era AOD peaks over Punjab & Haryana around 294 JD and takes to peak over NCR around 300 JD while post-2010 era witnesses around 305 and 311 JD respectively (Fig. 5b) indicating AOD plume reaches NCR with a time lag of 4–6 days as emitted from the hotspot region. In the Post-2010 era, NCR witnessed a sharp increase of ~ 23–26% in PM_2.5_ concentration and 4–6% in AOD during above PoSB_50_ while nominal increase of 8–10% in PM_2.5_ and 2–3% in AOD during PrSB_20_ over NCR region (from the baseline of background values i.e., NSB period). This substantiates the fact that with 32% increase of SB fires only in November intensifies aerosol PM emission over NCR by 23–26% affecting regional fog, visibility and health hazards.

## Discussion

**Figure 6:**
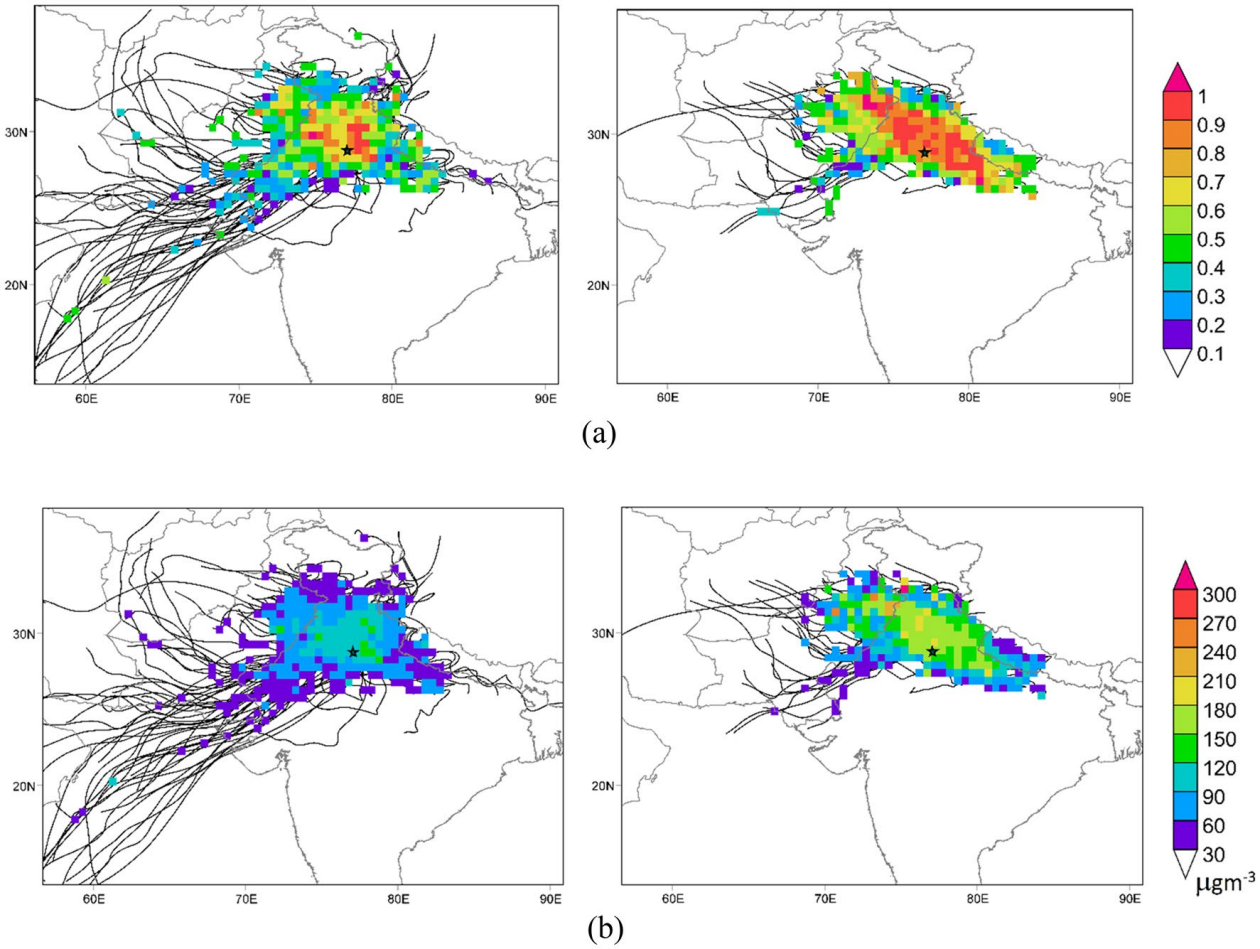
(**a**) Potential source contribution function (PSCF) and (**b**) Concentration weighted trajectories (CWT) analysis incorporating 5-day air mass back trajectory with PM_2.5_ concentration derived at receptor site Delhi (at 500 m agl) for PrM_SB_ (left) and PoM_SB_ (right) during 2015–2020. The location of the observation site (Delhi) is marked as star symbol. Maps are generated using opensource software MetInfoMap (http://meteothink.org).

Satellite based MODIS observations clearly capture heavy smoke and dust particulates emitted from SB which are dispersed & advected to downwind regions of IGP (hundreds of km) during PoM_SB_ (Supplementary Fig. 5b on right) due to synoptic meteorology (Supplementary Fig. 1b). From Potential Source Contribution Function (PSCF) and Concentration Weighted Trajectory (CWT) analysis, it is also evident that moderate to high PM_2.5_ concentration of 160–240 µgm^−3^ are advected upto central and parts of east IGP (Fig. 6) These north westerly winds transport the pollutants to NCR and get trapped owing to low boundary layer (< 500 m), low wind speed and direction, relative humidity and local meteorology which also prevents vertical mixing of pollutants^25,26^. Observation from Calipso shows the strong contribution of smoke aerosol that dominates the NCR and western IGP and present within 1 km of height (Supplementary Fig. 6). Similar results were also observed wherein smoke plumes originating from stubble fires are mostly concentrated between surface and below 1 km altitude and depending on the steady meteorological conditions take around 3–5 days to get transported in well homogenized manner over distance of 800 km in south eastern regions (NCR, IGP)^6,27^. It is observed from PSCF and CWT analysis (Fig. 6a,b) that PM_2.5_ plumes of low to moderate concentration (70–150 µgm^−3^) are observed during PrM_SB_ which are restricted to NCR and nearby regions and not much dispersed to central and eastern greater regions of IGP because of change in wind direction (southerly) (Supplementary Fig. 1b) and high boundary layer (> 1.5 km) (Supplementary Fig. 5a on right). The influence of aerosol dispersion is seen over East India and Eastern IGP during PrM_SB_ (Supplementary Fig. 5a on right) as it is influenced by the forest fires from North East India and Myanmar region (Supplementary Fig. 5a on left). Clearly there is no external advection of aerosol particulate from hotspot region over IGP during NSB period (Supplementary Fig. 5a,b on left). Noting that the area and production under rice and wheat in Punjab and Haryana did not change drastically after 2009 (production increase not more than 8%)^28^ which cannot be held responsible for increase in SB. Last 10 years has noticed an increase of 15.8% in SB fires (wheat and paddy SB combined), and on contrary a significant decrease of 4% in SB fires in the last 4 years (2016–2019 compared to 2011–2015) over the hotspot region. The impact of this reduction in SB fires during the last 4 years (2016–2019) is clearly evident in reduction in average PM_2.5_ concentration over Punjab, NCR and Delhi by 9.3%, 5.1% and 7.9% respectively (compared to 2011–2015). The number of severe PM_2.5_ days (> 250 µgm^−3^) has decreased by 7–9 days over NCR during this period. These results also collaborates with the 8% reduction in PM_2.5_ levels over Delhi^29^ (compared to annual average of 2015–2018). The reduction in SB fires and subsequently in PM_2.5_ is consequence of Government policies, sensitization and awareness amongst farmers towards the practice along with use of new agricultural technologies.

All the rivers flowing in Punjab and Haryana results from Himalayan snow melt, majorly contributing 58% and 50% to Punjab and Haryana river system respectively. The river system (Sutlej, Beas, Ravi, Chenab, Jhelum) in Punjab is spatially distributed with average flow rate ~ 6930 m^3^/s and the flow rate in Haryana region^30,31^ (Yamuna, Ghaggar) is ~ 2950 m^3^/s. This infers that there is good recharge of groundwater in Punjab compared to Haryana and hence, Punjab has comparatively good groundwater condition than Haryana region. The results from GRACE/GLDAS data have shown that Punjab is witnessing low depletion in GWS, than in Haryana (Supplementary Fig. 2a–d). This ascertains that despite having better GW recharge and good amount of rainfall, Punjab is also facing similar chronic water condition like Haryana. Therefore, the GRACE and CHIRPS data analysis validates this fact and features the utter plight of groundwater storage condition in both Punjab and Haryana region.

We also investigated the proportional air mass clusters originating from hotspot region and influencing & arriving at receptor site (Delhi)^32^. During PrM_SB_ around 40% of air mass clusters originating from hotspot region (north-west) are arriving at receptor site (New Delhi), 30% from oceanic region (south–west), 30% from western UP (north–east) and 10% from IGP (south–east) (Fig. 6a left). In PoM_SB_ around 70% of air mass clusters from hotspot region are arriving at receptor site (New Delhi), 20% from IGP, 5% from oceanic (Fig. 6a right). This clearly establishes the analysis that most of the pollutants and aerosols reaching Delhi and NCR originate from the hotspot SB region. Further, potential sources of PM_2.5_ observed over NCR were estimated using Potential Source Contribution Function (PSCF) and Concentration Weighted Trajectory (CWT) at a height of 500 m a.g.l as the aerosols and particulates are transported within the lower troposphere^6^. The high PSCF values (> 0.6) are considered as maximum probability sources to the receptor site and high CWT values (> 100 µgm^−3^) are the high strength sources (Fig. 6b). PSCF analysis confirms a strong probability (> 0.8) of PM_2.5_ sources to be located in North-west region (Fig. 6a right) and high PM_2.5_ air parcels are spread over the hotspot and nearby region (Fig. 6b). During PoM,_SB_ about 70% of PM_2.5_ trajectories are advected from hotspot region to the receptor site. We estimated the average contribution of Diwali event (considered ± 2 days from date of Diwali) to PM_2.5_ concentration during the period of 2016–2020 for daily data over 6 locations in Delhi (Anand Vihar, Lodhi Road, Mandir Marg, Punjabi Bagh, RK Puram and Shadipur). The analysis was done to see the rise of PM_2.5_ levels during Diwali days with respect to the PoSB_50_ (non-Diwali) period. The background PM_2.5_ pollution level during 2016–2020 is considered for PoSB_50_ period. The analysis reveals that the average contribution of Diwali adds 32 µgm^−3^ to PM_2.5_ of days which is similar to findings that two days of Diwali (during 2013–2017) adds 40 µgm^−3^ to PM_2.5_ concentration over Delhi^33^. This influx from hotspot region abruptly increases PM_2.5_ concentration over NCR (250–300 μgm^−3^) especially Delhi (350–400μgm^−3^). The estimates also show that during 2010–2020, the PM_2.5_ CWT reaching Delhi  have increased at a rate of 1% (PrM_SB_) and 3% (PoM_SB_) per year.

The analysis shows that NCR air quality is not only affected from stubble burning but also a combined effect of synoptic low and steady winds, low boundary layer, favorable wind direction and stable atmosphere. In order to improve the air quality over NCR and northern India, there needs to be long term and short-term steps to be taken. Government of India under National Action Plan for Climate Change (NAPCC) had enforced National Policy for Management of Crop residue (NPMCR) in 2014. Government is implementing ways to help farmers through use of subsidizing machinery equipment to cut stubble, promote diversified utilization of stubble for in-situ crop management, many industrial application and other uses. While the positive effect is being visible by way of slight improvement in air quality over NCR but will take time for substantial improvement. Under the short-term step, adopt rainwater harvesting & other water conservation technologies for optimal use of available water resources, renovation & use of village ponds, restore field distributaries & increase irrigation area under canal system, shifting from monoculture to diversified crop pattern and adoption to replace current rice variety with shorter duration rice varieties. This would make harvest 10–15 days (in September month) earlier than current practice period. During this time, the winds are moderate (3–4 m/s), substantial high temperature (5–6 C more than in November) and unfavorable wind direction (westerly to south-westerly) which does not transported and advect smoke to the NCR and adjoining areas in Northern India thereby reducing the number of high polluted episodes (PM_2.5_ > 200 µgm^−3^) and not coinciding with the festive period^34^. This will also help in reduction in contribution of stubble burning share to PM_2.5_ by 50%^34^.

## Conclusion

Using long term satellite and ground measurements, we have investigated the trends in stubble fires, associated particulate matter pollution over NCR, downwind regions and linkage with groundwater storage. The present water preservation act was implemented to preserve the groundwater by delaying the paddy nursery and transplantation. However, this policy has led to delay in harvest & shrinking of harvest window with increased SB fire occurrences (15.8%) and shift in SB by 8–10 days (wheat) and 10–12 days (Paddy). The particulate matter pollution associated with SB fires coincides with cultural festivals in India thereby increased pollution over North India. There is no improvement in groundwater storage change after 2013 and superfluous usage for irrigation is leading to depletion of groundwater storage at a rate of 29.2 mmyr^−1^ over these regions and the GW act is marginalized in preventing the depletion of groundwater storage. Almost 21% increase in Paddy SB fires over a decade added additional 23–26% of annual PM_2.5_ concentration over NCR. This high influx of aerosol particulate matter over NCR and downwind regions (particularly during PoM_SB_) is deteriorating air quality in north India to as much of 4–5 times the permissible Indian standard. However, due to implementation of NPMCR policy & awareness programs among farmers by Government, the SB fires incidents and related pollution over NCR are on marginal decline (5%) over the last 4 years. It is envisaged that the approach and steps proposed in this study could be potential options for policy planners. This in turn will have positive impacts on air pollution with reduced mortality and morbidity rates.

## Methods

### Fire data collection and analysis

MODIS Thermal Anomalies/Fire locations—Collection 6 daily data of Aqua and Terra combined (MCD14DL) (distributed by LANCE FIRMS) having spatial resolution of 1 km x 1 km and a swath of 1200 km is used. Daily data from 2002–2020 for PrM_SB_ and PoM_SB_ was collected and classified into high confidence (> 80%), medium confidence (30–80%) and low confidence (< 30%) data. In order to capture the SB events where the fires are generally small (< 1 hectare of agricultural field) data > 30% confidence level data was used so as to capture smallest fire event. The fire density is calculated using MODIS fire data at 1 km resolution. The Climate modelling grid (CMG) fire data are gridded statistical summaries of fire pixel information intended for use in regional and global modelling and other large-scale studies and are generated at 0.25 deg. resolution for the time periods. Hence, for estimating regional fire density we used at standard 0.25 deg. square grid. For anlaysis, Pre-Monsoon (PrM_SB_) (i.e., wheat stubble) is considered from 1April–31May every year while Post-monsoon (PoM_SB_) (i.e., paddy stubble) is considered from 1 October–30 November every year. PrSB_20_ is the sub-set of SB period where the burning is substantial (*i.e*., fires > 20 day^−1^ during PrM_SB_) and PoSB_50_ period (fires > 50 day^−1^ during PoM_SB_). NSB is ± 30 days from the PrM_SB_ or PoM_SB_ period.

### Aerosol optical depth

Daily Level-2, Collection-6 data (https://ladsweb.modaps.eosdis.nasa.gov/) MODIS/Aqua (MOD04_3k) and MODIS/Terra (MYD04_3k) AOD with a spatial resolution of 3 km and swath resolution approximately 2330 km wide was used to study the aerosol distribution in the region. The expected error in AOD is around ± 0.05^35^. For analysis, daily data has been collected, processed and analyzed for PrM_SB_, PoM_SB_ and non-SB period during 2002–2020.

### Air quality data

For analyzing the effect of SB on particulate concentration (PM_2.5_) over NCR, the quality assured ground PM_2.5_ continuous data available for more than 4 years was considered (https://app.cpcbccr.com/ccr/#/caaqm-dashboard/caaqm-landing/caaqm-data-availability) is found over 19 stations in NCR region (Anand vihar, Dilshad Garden, Lodhi Road, Mandir Marg, NSIT Dwarka, DU North Campus, Punjabi Bagh, RK Puram, Shadipur, Sirifort, Mathura road, DTU, IGI Airport, ITO and Pusa in Delhi; Sec 16A in Faridabad; Vikas sadan in Gurugram; Vasundara in Ghaziabad; and Sec 62 in Noida). The standard error mean of the data is 2.68. The data quality control and calibration of the instruments is being done regularly as per the protocols laid be CPCB (https://cpcb.nic.in/quality-assurance-quality-control/). The CPCB (Central Pollution Control Board), Government of India monitors the level of pollutants and trace gases at 573 stations in 240 cities which covers 26 states and 5 union territories (http://cpcbenvis.nic.in/airpollution /monetoring.htm).

### Aerosol subtype data

CALIPSO (Cloud–Aerosol Lidar and Infrared Pathfinder Satellite Observations) was launched in 2006 at an altitude of 705 km by by NASA Langley Research Centre in collaboration with the French Space Agency CNES. The primary goal of CALIPSO is to investigate the vertical distribution of aerosol and clouds and its impact of Earth’s climate and air quality^36^. In this study, CALIPSO Level 2 product (version 4.10) on Aerosol subtype is used to identify the smoke aerosols position and its vertical extent along the overpass path. A typical post-monsoon CALIPSO overpass over the smoke affected region (NCR) on 3rd November 2018 is utilized in this analysis.

### Surface winds

The CAMS reanalysis data is used for estimating the surface winds (10 m) during PrM and PoM. It is the latest global reanalysis dataset which consists of 3-D time consistent atmospheric composition fields (aerosols, chemical species and greenhouse gases). The reanalysis data is produced vertically at 60 hybrid sigma/pressure levels using 4Dvar data assimilation in CY42R1 of ECMWF’s Integrated Forecast System^37^. 10 m u and v component of wind is taken at 0.25 × 0.25-degree grid for plotting wind direction and magnitude over IGP.

### Groundwater storage

Gravity Recovery and Climate Experiment (GRACE), dataset of RL06, Level 3 GRACE SH (average of CSR, JPL and GFZ data monthly solution)(http://grace.jpl.nasa.gov), is used to estimate monthly Terrestrial Water Storage (TWS), expressed in equivalent water thickness in cm with a spatial resolution of 1° × 1°. Global Land Data Assimilation System (GLDAS 2.0-NOAH) is used to estimate monthly total soil moisture (TSM), canopy water storage (CWS) and snow water equivalent (SWE) (Kg m^−2^) and surface water (storm water) (SW) with a spatial resolution of 1° × 1° (https://ldas.gsfc.nasa.gov/data). GRACE based Terrestrial Water Storage (TWS) is an integrated measure of groundwater storage (GWS) and GLDAS_total_. The change in groundwater storage rate (mm/year) is estimated from GLDAS_total_ which is a measure of difference the aggregate of (TSM, SWE, SW and CWS) and the mean aggregate of (TSM, SWE, SW and CWS). We have estimated the groundwater mass loss (km^3^) by multiplying the groundwater storage rate with the area calculated for both Punjab and Haryana region. In this study, the groundwater storage is estimated in three time periods; 2002–2009, 2010–2013 and 2014–2017. Central Ground Water Board (CGWB) annually monitors around 15,000 groundwater observation wells in India (3500 piezometers of 40–100 m depth and rest dug wells/tube wells) four times (January, April/May, August and November). The well dataset (391 wells, measured in mbgl) of Central Ground Water Board (CGWB 2002 & 2012), Government of India has collected quarterly (pre-monsoon and post-monsoon) for the period 2005–2009, 2010–2013 & 2014–2016. Out of total 15,000 wells located in India, 391 wells are located in Punjab and Haryana regions. Factors like temporal gaps and missing data can create discrepancies in the well datasets. Hence, it is imperative to select suitable wells for pre-processing so that requisite and accurate results can be achieved^38,39^. In the study, all 391 well data was used. GRACE derived groundwater storage and well groundwater level data were compared using Pearson coefficient. To make sure that groundwater storage depletion rate has not been affected by any other climatic factors, we investigated the TRMM (Tropical Rainfall Measuring Mission) rainfall data for these three consecutive periods (2003–2009, 2010–2013, 2014–2016). The average precipitation rate (cm) estimated over Haryana and Punjab region for the above time period is 53.5 cm, 61.2 cm and 54.1 cm respectively. The ground recharge rate for 2010–2013 may have increased but overall, over the years, there is seen a decrease in rate of replenishment due to overall irrigation practices.

Climate Hazard group Infrared Precipitation with Stations (CHIPRS) is a quasi-global, reliable and contemporary dataset that is highly helpful in identifying early warning signatures like seasonal drought etc. It uses the information from satellite for representing the rain gauge data distributed sparsely in different locations, features 0.05° × 0.05° spatial resolution and measures on daily, monthly and pentad time scales. This data utilizes the TRMM (Tropical Rainfall Measuring Mission Multi-Satellite Precipitation) data of TMPA3B42v7 for measuring the rainfall estimates derived from CCD (Cold Cloud Duration) globally. It uses an approach of smart interpolation and cofunction with anomaly derived from climatology of high resolution. CHIRPS uniquely produces two products in 2 phase processes; in first phase it yields rainfall product with delay in two days and the gauge data from World Meteorological Organization’s Global Telecommunication System (GTS) amalgated with the rainfall estimates (pentad) derived from CCD. While in the second phase the final product is obtained with delay in 3 weeks, and the pentad/monthly best available rainfall estimates derived from CCD is merged with the station data (derived monthly/pentad^40^. The Standardized Precipitation Index (SPI) uses CHIRPS monthly datasets for shedding light on drought conditions at various scales. It is an emerging method for estimating the drought index, and was formulated by the integrated effort of Tom Mckee, Nolan Doesken and John Kleist of Colorado Climate Center^41,42^. They distributed drought condition into several classes depending on its respective SPI value (Supplementary table 3)**.** In this study we used SPI calculation on 6-month time scale (SPI-6) using CHIPRS (https://www.chc.ucsb.edu/data/chirps), as this particular time scale is sensitive to the changes in rainfall (both seasonal and inter-seasonal). The 6-month SPI calculation includes current month precipitation and then compares with the precipitation of the exact six-month period of previous years. For instance; the SPI value at April–September month is compared with total precipitation of the same six months (April–September) period from previous years.

### Air mass back trajectory and source apportionment

We modelled 5-day backward air mass trajectories at receptor site Delhi at 500 m a.g.l separately for PrM_SB_ and PoM_SB_ during 2011–2020 using HYSPLIT (Hybrid Single Particle Lagrangian Integrated Trajectory) model (HYSPLIT V5) using NCEP/ NCAR reanalysis archived data (ftp://arlftp.arlhq.noaa.gov/pub/archives/reanalysis). To investigate the sources, the air mass trajectories were computed for clusters. The PM_2.5_ emitted source regions are identified using Concentration Weighted Trajectory (CWT) algorithm by incorporating actual ground measured PM_2.5_ data (Source PM_2.5_ data: CPCB). Further, to understand dispersion and advection of the SB emissions to influence regions, we used Potential Source Contribution Function (PSCF) which is a receptor model that incorporates meteorological information in its analysis scheme to produce a probability field that can be used to determine areas of the potential source contribution.

### Statistical tests

Non-parametric Mann–Kendall test was conducted for the trend analysis with statistical significance established at p < 0.05. Pearson’s coefficient was used to measure the correlation between satellite observed and ground in-situ data.

## Supplementary Information

Supplementary Information.
