# Author Correction: Long term influence of groundwater preservation policy on stubble burning and air pollution over North-West India

**DOI:** 10.1038/s41598-022-06980-4

**Published:** 2022-02-11

**Authors:** Yogesh Kant, Prakash Chauhan, Aryan Natwariya, Suresh Kannaujiya, Debashis Mitra

**Affiliations:** Indian Institute of Remote Sensing (IIRS), ISRO, Department of Space, Govt. of India, 4-Kalidas Road, Dehradun, India

Correction to: *Scientific Reports* 10.1038/s41598-022-06043-8, published online 08 February 2022

The original version of this Article contained an error in the Results, under the subheading ‘Long term variation in stubble fires’,

“Monthly fire counts of post-2010 era, reveal that the April SB fires have decreased (0.04% yr^−1^) and fires in May have increased (0.065% yr^−1^ while decrease of stubble fires in October (2.1% yr^−1^) and a significant jump in November (3.2% yr^−1^) (Fig. 1a,b).”

now reads:

“Monthly fire counts of post-2010 era, reveal that the April SB fires have decreased (0.4% yr^−1^) and fires in May have increased (0.65% yr^−1^ while decrease of stubble fires in October (2.1% yr^−1^) and a significant jump in November (3.2% yr^−1^) (Fig. 1a, b).”

The original Article has been corrected.

