# Supplementary material for: Long term influence of groundwater preservation policy on stubble burning and air pollution over North-West India
Source: Sci Rep. 2022 Feb 8;12:2090. doi: 10.1038/s41598-022-06043-8 (PMC8825838; doi:10.1038/s41598-022-06043-8)
Supplement: Supplementary file 1 — Supplementary Information. [file 41598_2022_6043_MOESM1_ESM.docx]

**Long term influence of Ground water preservation policy on stubble burning and air pollution over North-West India**

**Yogesh Kant, Prakash Chauhan^*^, Aryan Natwariya, Suresh Kannaujiya and Debashis Mitra**

**Indian Institute of Remote Sensing (IIRS), ISRO**

**Dept. of Space, Govt. of India**

**4-Kalidas Road, Dehradun**

**Supplementary Figures**

**
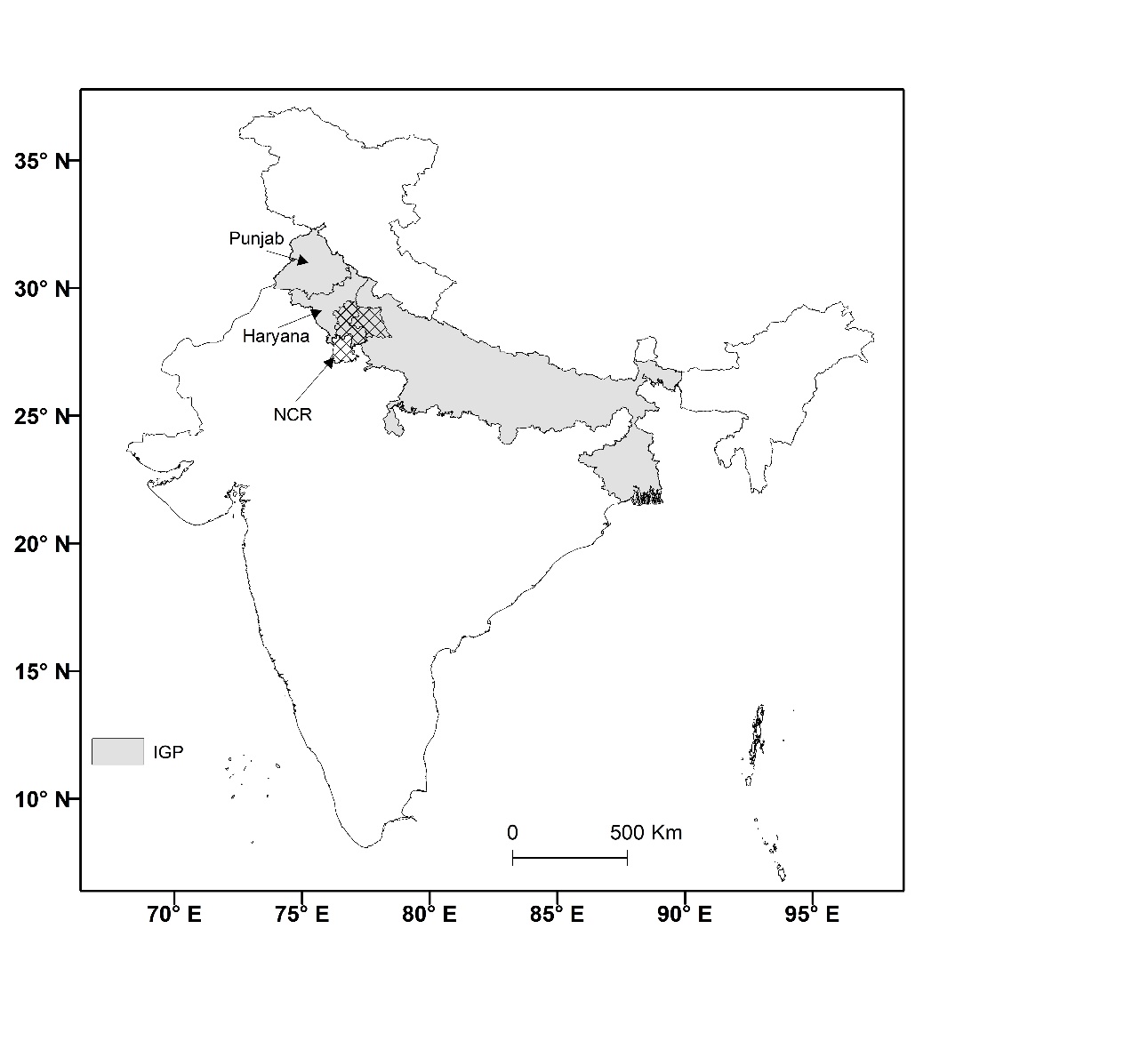
**

**(a)**


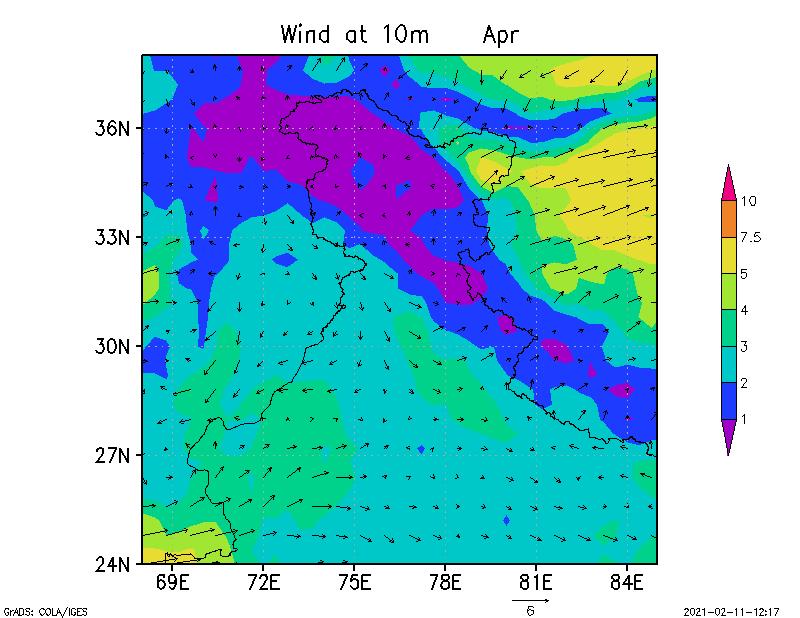

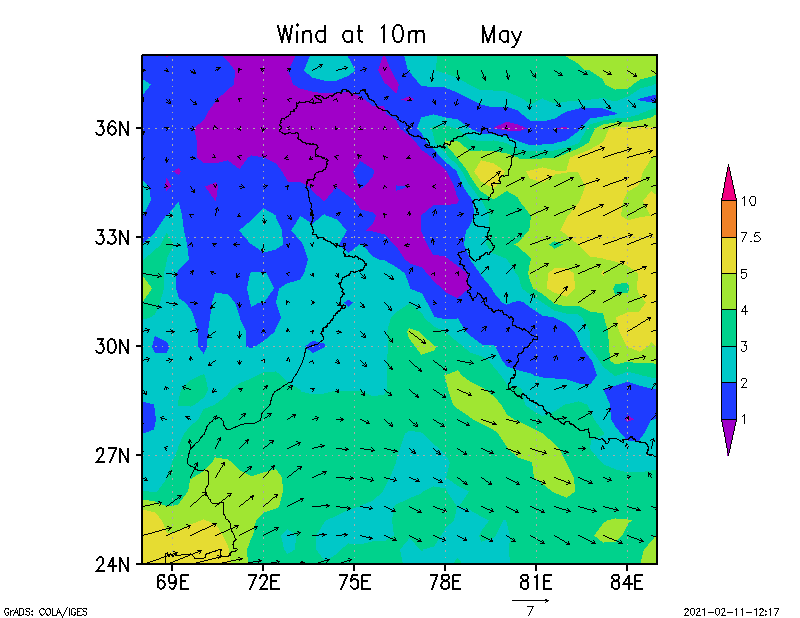

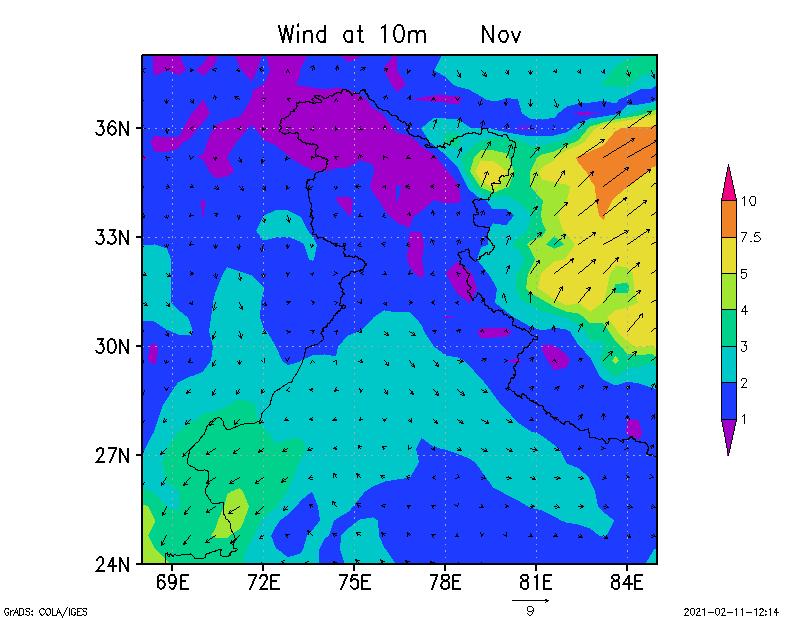

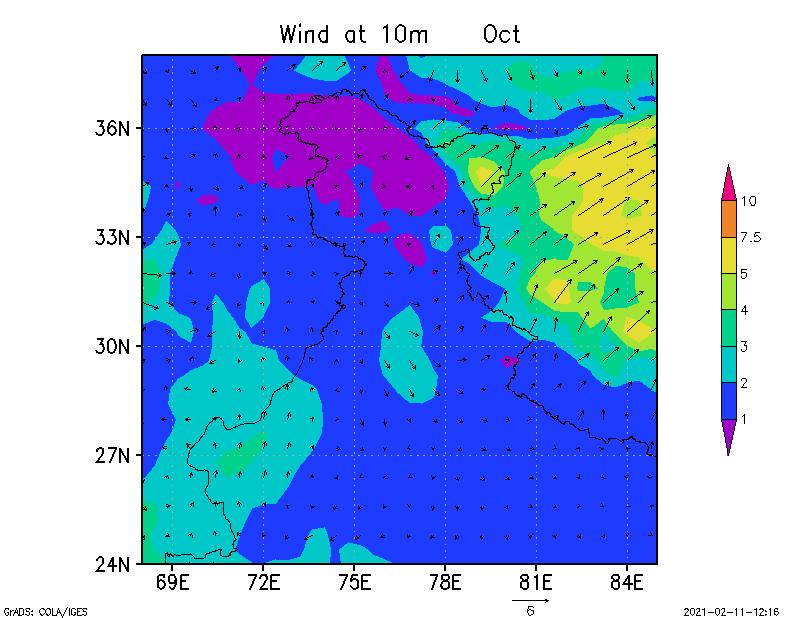

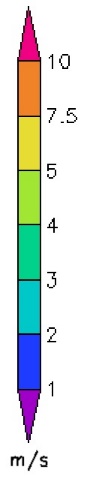


**(b)**

Supplementary Fig. 1: (a) Map showing Punjab, Haryana, NCR and IGP; (b) surafce winds at 10m height (averaged for 5 years) derived from CAMS ECMWF for April-May and October-November over IGP. The figures are generated using open source software GrADS version 2.0.2 (http://cola.gmu.edu/grads/downloads.php).


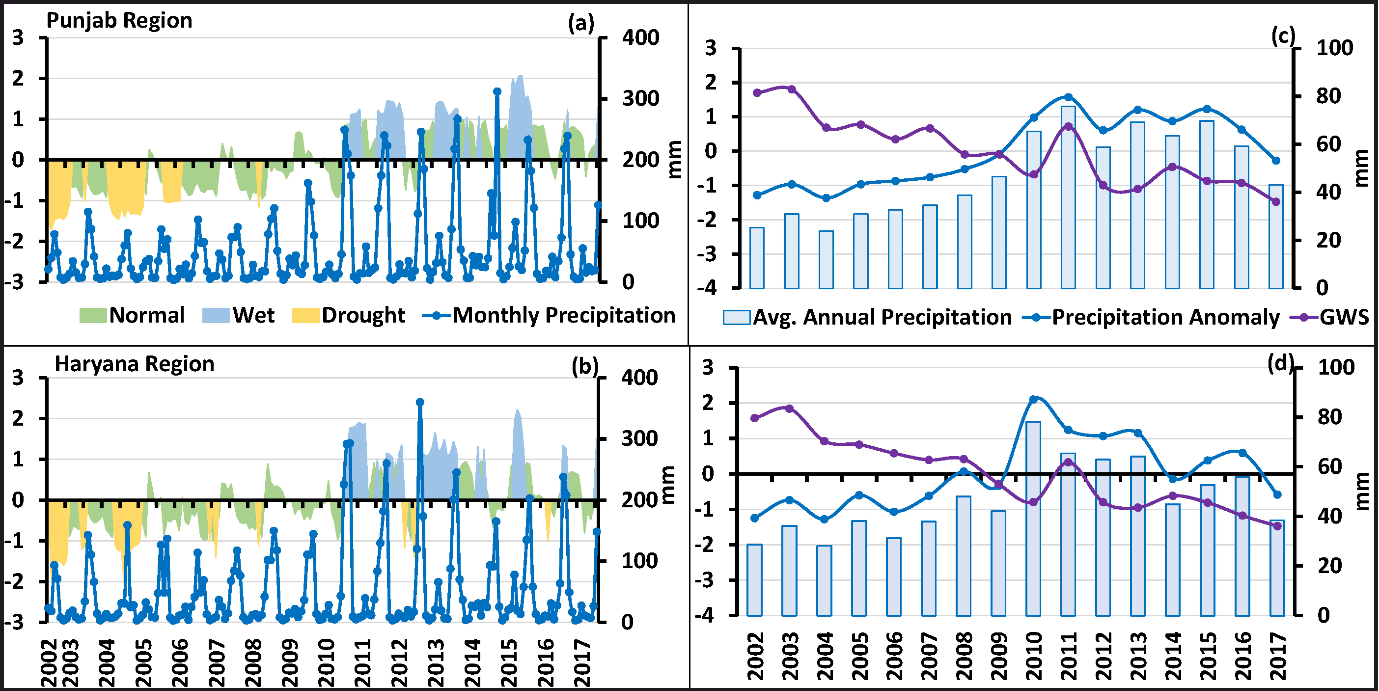


Supplementary Fig. 2 (a) & (b) Time series plotted on SPI variation in Punjab and Haryana regions, respectively. (c) & (d) Correlating the estimated GWS calculated from the GRACE dataset with the average annual precipitation and precipitation anomaly of Punjab and Haryana region, respectively.

*
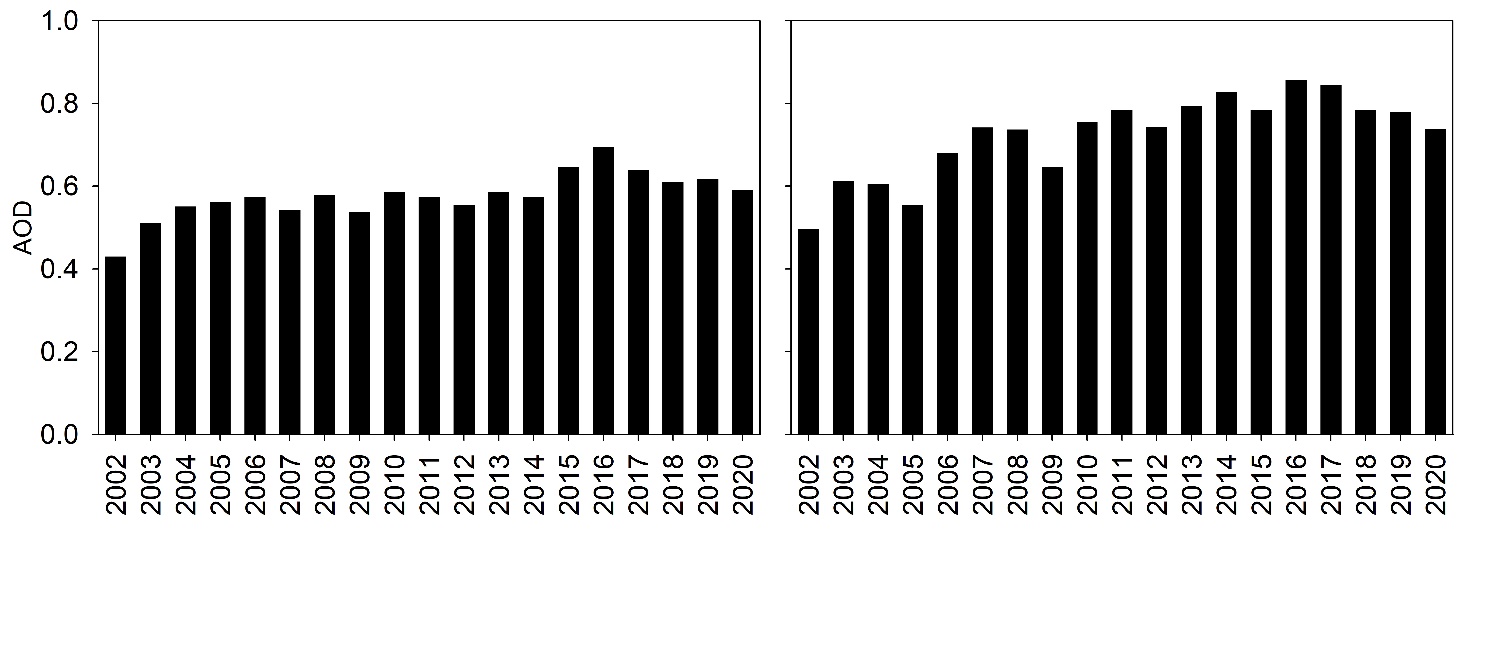
*

(a) (b)

Supplementary Fig. 3: Time-series of AOD (a) PrM_SB_ and (b) PoM_SB_ over NCR during 2002-2020

(a) (b)


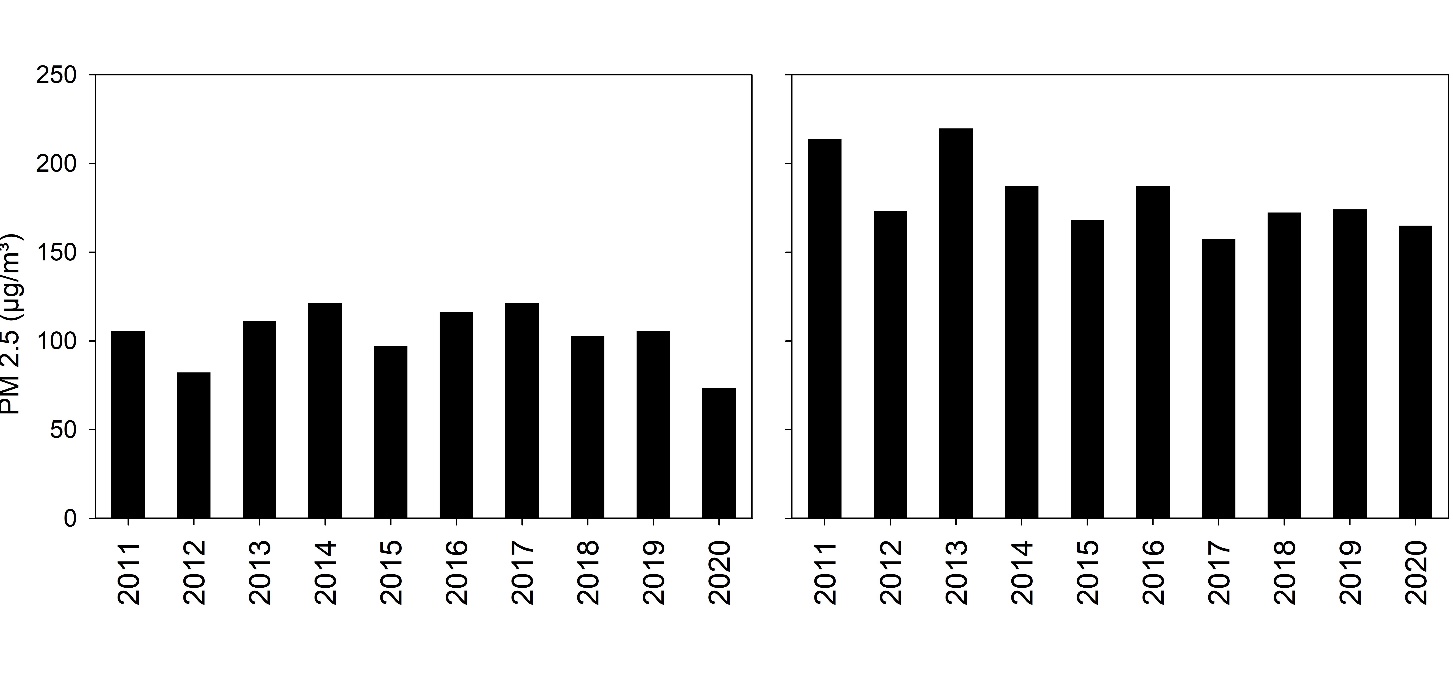
Supplementary Fig. 4: PM_2.5_ concentration during (a) PrM_SB_ and (b) PoM_SB_ over NCR during 2011-2020


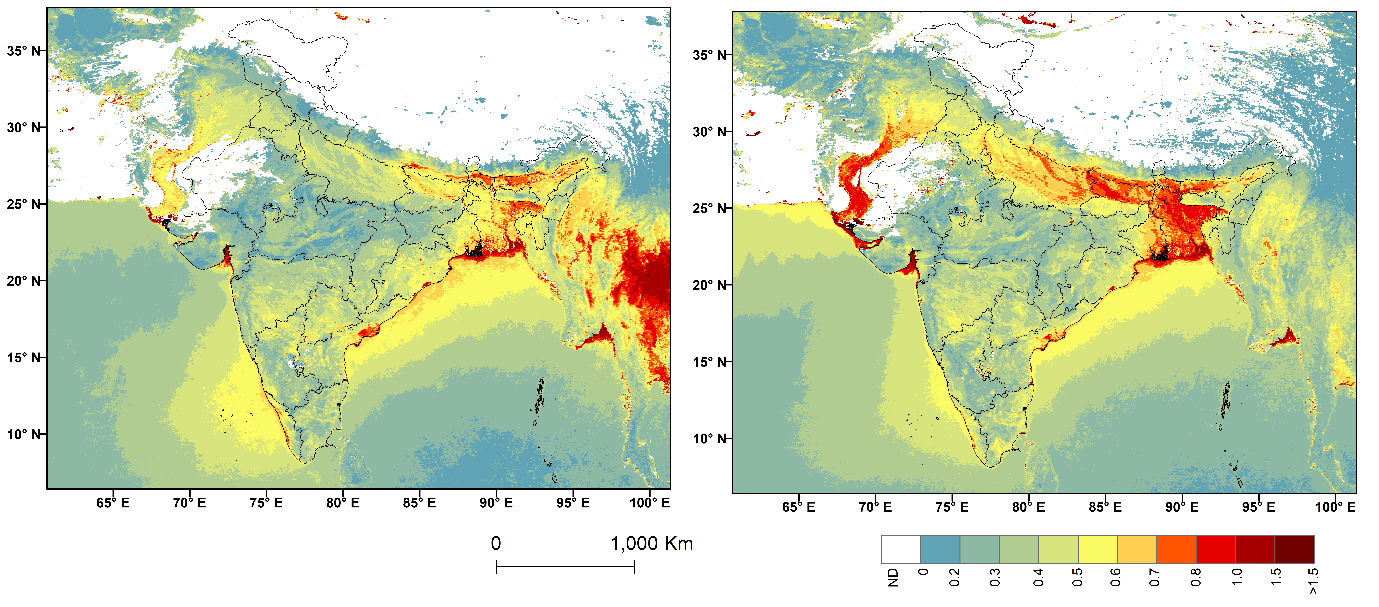


(a)


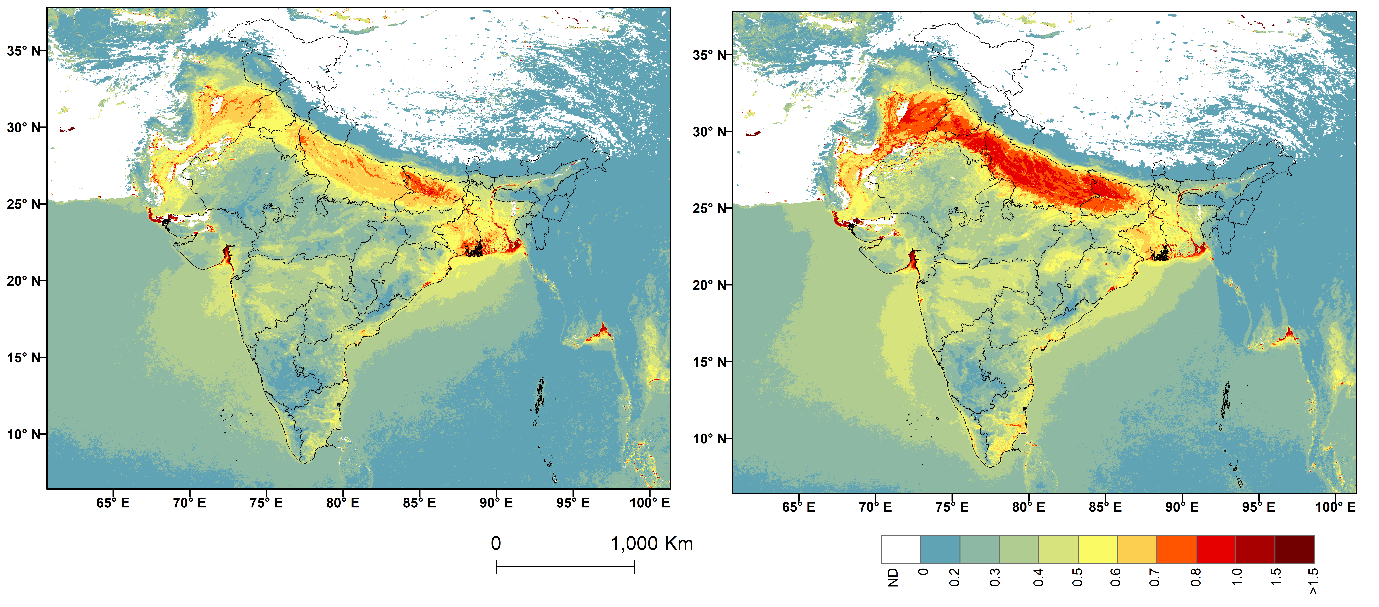


(b)

Supplementary Fig. 5: Daily averaged MODIS AOD for (a) PrSB_20_, (b) PoSB_50_ period (right) and NSB period (left). Maps are generated using licensed software ArcGIS version 10.6.

**
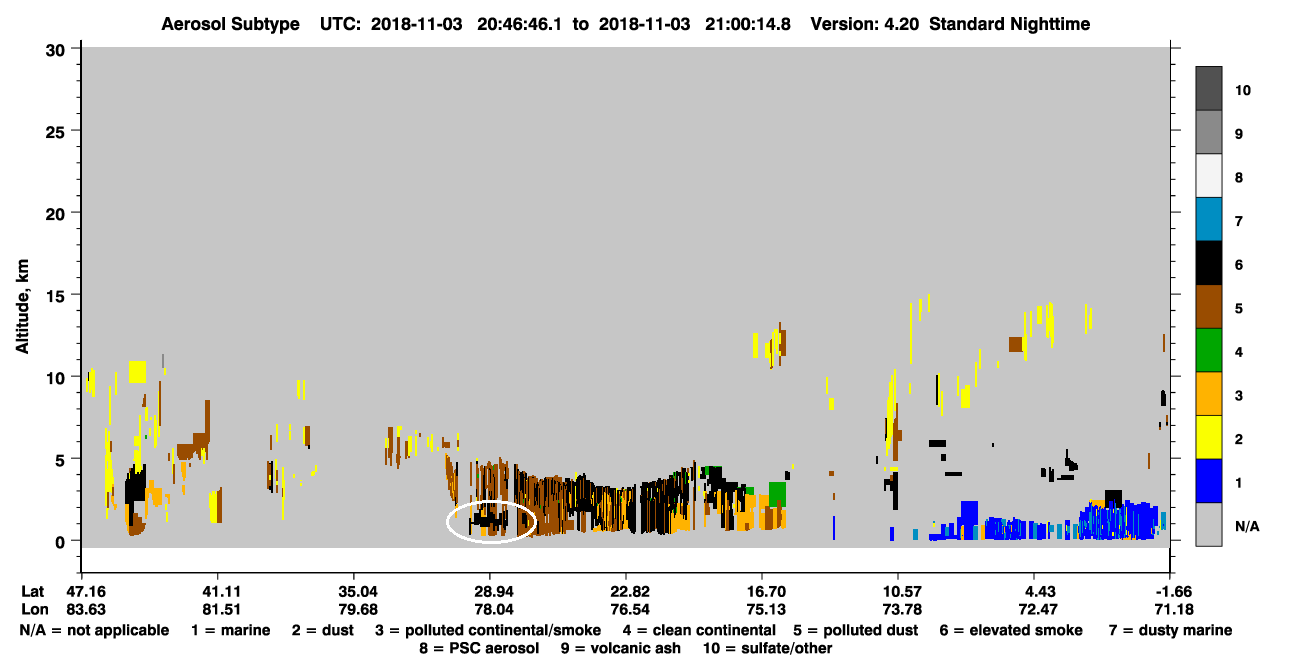
**

Supplementary Fig. 6: CALIPSO observation on Aerosol subtypes over pass near NCR region (circle denotes NCR region) on 3^rd^ November 2018. The image is downloaded from open source <https://www-calipso.larc.nasa.gov/products/>

**Supplementary Table 1**- The estimated groundwater storage rate and mass loss due to GWS depletion observed from the GRACE Dataset.

| Region | Groundwater Storage Rate (mm yr^-1^) | | |
| --- | --- | --- | --- |
|  | **2002-2009** | **2010-2013** | **2014-2017** |
| Punjab | -22.87±2.81 | -22.70±5.64 | -26.47±6.25 |
| Haryana | -27.65±3.78 | -19.49±5.22 | -32.09±10.59 |
|  | **GWS mass loss (km^3^)** | | |
| Punjab | -10.32 | -5.11 | -5.30 |
| Haryana | -11.71 | -3.17 | -5.83 |

**Supplementary Table 2**- Groundwater level rate estimated from dug well and tube well dataset (Source: CGWB).

| Region | Groundwater level Rate (m yr^-1^) | | |
| --- | --- | --- | --- |
|  | **2005-2009** | **2010-2013** | **2014-2016** |
| Punjab | -2.28±0.67 | -1.38±0.59 | -3.20±1.12 |
| Haryana | -2.90±0.56 | -1.27±0.77 | -4.51±1.50 |

Supplementary Table 3- Drought classification on the basis of SPI value (modified after Mc Kee et al., 1993)

| Drought Category | SPI Value |
| --- | --- |
| Extremely wet | >2.00 |
| Very wet | 1.50 to 1.99 |
| Moderate wet | 1.00 to 1.49 |
| Normal | -0.99 to 0.99 |
| Moderate drought | -1.0 to -1.49 |
| Severe drought | -1.50 to -1.99 |
| Extremely drought | <-2.00 |

Supplementary Table 4- Available data range for CPCB PM measuring stations

| **S.No.** | **Station Name** | **Data Range** |
| --- | --- | --- |
| 1 | Anand vihar | July, 2012- Dec,2020 |
| 2 | Dilshad Garden | Jan,2009- Dec,2020 |
| 3 | Lodhi Road | Jan,2014- Dec,2020 |
| 4 | Mandir Marg | April,2011-Dec,2020 |
| 5 | NSIT Dwarka | Jan,2009- Dec,2020 |
| 6 | DU North Campus | Sep,2015- Dec,2020 |
| 7 | Punjabi Bagh | April,2011-Dec,2020 |
| 8 | RK Puram | April,2015- Dec,2020 |
| 9 | Shadipur | Jan,2009- Dec,2020 |
| 10 | Sirifort | Jan,2007- Dec,2020 |
| 11 | Mathura road | Aug,2017- Dec,2020 |
| 12 | DTU | Nov,2016- Dec,2020 |
| 13 | IGI Airport | Aug,2017- Dec,2020 |
| 14 | ITO | Sep,2006- Dec,2020 |
| 15 | Pusa | Sep,2019- Dec-2020 |
| 16 | Sec 16A (Faridabad) | Sep,2010- Dec,2020 |
| 17 | Vikas sadan (Gurugram) | Dec, 2015- Dec,2020 |
| 18 | Vasundara (Ghaziabad) | Sep,2015- Dec,2020 |
| 19 | Sec 62 (Noida) | Sep,2017-Dec,2020 |
